# Comparative analysis of *draper* mutant alleles and RNAi expression systems in the ovary and brain of *Drosophila melanogaster*

**DOI:** 10.1093/g3journal/jkag040

**Published:** 2026-02-16

**Authors:** Guangmei Liu, Pamela Yang, Trung Le, Jerald Shin, Cheng Yang Shi, Emily Budhram, Kimberly McCall

**Affiliations:** Department of Biology, Boston University, 5 Cummington Mall, Boston, MA 02215, United States; Department of Biology, Boston University, 5 Cummington Mall, Boston, MA 02215, United States; Department of Biology, Boston University, 5 Cummington Mall, Boston, MA 02215, United States; Department of Biology, Boston University, 5 Cummington Mall, Boston, MA 02215, United States; Department of Biology, Boston University, 5 Cummington Mall, Boston, MA 02215, United States; Department of Biology, Boston University, 5 Cummington Mall, Boston, MA 02215, United States; Department of Biology, Boston University, 5 Cummington Mall, Boston, MA 02215, United States

**Keywords:** *draper*, phagocytosis, ovary, neurodegeneration, gene expression system

## Abstract

The phagocytic receptor Draper (Drpr) mediates clearance of apoptotic cells in *Drosophila melanogaster*, yet how distinct *drpr* alleles and RNAi constructs differ in efficiency and phenotypic outcomes has not been systematically compared. Here, we evaluate multiple *drpr^RNAi^* lines across UAS/GAL4, QUAS/QF2, and LexA/LexAop systems, alongside a newly generated CRISPR allele (*drpr^CR1^*). Immunostaining confirmed efficient protein knockdown for all RNAi lines, while qRT-PCR revealed variable transcript reduction, with short hairpin (SH) constructs more effective in glia and long hairpin (LH) constructs likely acting at the translational level. Despite these differences, all RNAi lines caused strong phenotypes with persisting nurse cell nuclei in the ovary, and apoptotic cell persistence and neurodegenerative vacuoles in the brain, with SH constructs producing more severe defects. The newly generated *drpr^CR1^*, which selectively deletes exons 5 to 6, abolished full-length Drpr-I while preserving shorter isoforms. Unlike the widely used *drpr^Δ5^* allele, *drpr^CR1^* uncouples ovarian and brain phenotypes: Both alleles display ovarian defects, but *drpr^CR1^* shows markedly reduced neurodegeneration compared to *drpr^Δ5^*. Together, our findings reveal construct- and allele-specific differences in RNAi knockdown and *drpr* isoform function, demonstrating that full-length Drpr is indispensable for ovarian cell clearance but less critical for neurodegeneration.

## Introduction

The *Drosophila melanogaster* ortholog of the CED-1 engulfment receptor in *Caenorhabditis elegans*, Draper (Drpr), plays a critical role in the recognition and clearance of apoptotic cells and cellular debris, a process fundamental to tissue homeostasis. Initially identified in glial clearance of dying neurons in the *Drosophila* brain (Freeman et al. 2003), Drpr is now recognized as a central mediator of phagocytosis by nonprofessional phagocytes in the ovary, testis, and nervous system (Etchegaray et al. 2012; Han et al. 2014; Zohar-Fux et al. 2022), in addition to playing an essential role in hemocytes, the professional phagocytes of *Drosophila* (Manaka et al. 2004). In neural and gonadal contexts, Drpr activates c-Jun N-terminal kinase (JNK) signaling to promote engulfment (Awasaki et al. 2006; Ziegenfuss et al. 2008; Etchegaray et al. 2012; Serizier et al. 2022; Zohar-Fux et al. 2022).

To dissect Drpr's tissue-specific roles and downstream signaling pathways, a range of genetic tools have been developed. The most widely used include the *drpr*^Δ5^ allele and RNA interference (RNAi) transgenic lines, each offering distinct advantages. RNAi-inducing constructs in *Drosophila* may use long double-stranded RNAs (dsRNAs) or small hairpin RNAs (shRNAs). The *drpr^Δ5^* allele, which deletes the promoter and part of the 5′ UTR of the gene (Freeman et al. 2003), enables putatively complete loss of Drpr function and has been instrumental in uncovering its involvement in neurodevelopmental debris clearance, germline cell degradation, phagoptosis of germ cells, muscle degeneration, and age-dependent neurodegeneration (Freeman et al. 2003; Etchegaray et al. 2012, 2016; Draper et al. 2014; Timmons et al. 2016; Zohar-Fux et al. 2022). On the other hand, RNAi-mediated knockdown using binary genetic systems such as UAS/GAL4, QUAS/QF and LexAop/LexA systems allows spatiotemporal control of gene silencing. Most commonly, a long dsRNA construct has been used to knock down *drpr* with the UAS/GAL4 system. This RNAi line has been widely used to study cell-type-specific functions of Drpr in tissues such as glia, follicle cells, and epidermal cells (MacDonald et al. 2006; Etchegaray et al. 2012; Han et al. 2014). Loss of Drpr in glia results in the accumulation of apoptotic cells, retention of degenerating axons, and age-dependent neurodegeneration, while its absence in follicle cells leads to persisting nurse cell (PNC) nuclei, indicating defective cell clearance (Etchegaray et al. 2012, 2016; Logan et al. 2012; Timmons et al. 2016; Elguero et al. 2023). In epidermal cells, Drpr is required for engulfing degenerating dendrites during development; its loss leads to the retention of dendritic debris (Han et al. 2014). Additional RNAi lines have been developed but not used as widely.

These genetic tools vary in genetic background, knockdown efficiency, and molecular design, including differences in RNAi construct type, targeted transcript region, and insertion sites. Furthermore, since the *drpr^Δ5^* allele disrupts only the noncoding region (Freeman et al. 2003), it may not fully eliminate gene activity, potentially allowing residual Drpr function. To address this, newer alleles generated via CRISPR/Cas9 genome editing offer a more definitive knockout by precisely targeting coding sequences, ensuring a complete loss of function. However, no study to date has systematically compared the performance, phenotypic outcomes, and tissue specificity of these various *drpr* loss-of-function and knockdown tools. This lack of standardization presents a major challenge for interpreting results and making comparisons across studies—especially in an era of increasingly refined genetic dissection. Moreover, the use of more than 1 binary expression system (e.g. UAS/GAL4 and LexAop/LexA) is particularly valuable in tissues where experiments involve 2 distinct cell types that need to be manipulated separately.

In this study, we generated a new *drpr* mutant using CRISPR/Cas9 technology and performed a comprehensive molecular and phenotypic comparison of commonly used *drpr* mutant and RNAi lines in different binary expression systems. By assessing gene deletion or knockdown efficiency, tissue specificity, and phenotypic impact in both the brain and ovary, we aim to identify the strengths and limitations of each approach. Our results provide a valuable resource for the field and a framework for choosing the most appropriate genetic strategy for future investigations of Drpr-mediated phagocytosis.

## Materials and methods

### Fly stocks and husbandry

All fly lines were raised at 25 °C on standard cornmeal and molasses food. Flies for all experiments were 5 to 9 d old except for neurodegeneration examination. For neurodegeneration assessment, flies were aged to 40 d. Aging flies were transferred to fresh food every 3 to 4 d. The following fly strains were used: *w^1118^* [Bloomington *Drosophila* Stock Center {BDSC} #5905] (Ryder et al. 2004), *drpr^Δ5^* (Freeman et al. 2003), *drpr^CR1^* (generated in this study), *ctrl1* (generated in this study), *GR1-Gal4* (Goentoro et al. 2006), *tj-Gal4* (Rivera et al. 2025), *UAS-lexA^RNAi^ (*BDSC #67947*)*, *UAS-drpr^RNAi^* [long hairpin {LH}, BDSC #67034] (MacDonald et al. 2006), *UAS-drpr^RNAi^* [short hairpin {SH}, BDSC #36732], *UAS-mCD8::GFP* (BDSC #32189), *lexAop-drpr^RNAi^* (Coutinho-Budd et al. 2017), *tub-lexA* (BDSC #66686) (Lai and Lee 2006), *lexAop-mCD8::GFP* (BDSC #32203), *tub-QF2/TM6B* (BDSC #66478), *repo-Gal4/TM3* (BDSC #7415), *repo-lexA* (BDSC #97535), *repo-QF2* (BDSC #66477), *QUAS-white^RNAi^*, *QUAS-drpr^RNAi^*, *QUAS-mCD8::GFP* (BDSC #30001). Unless specified, the RNAi lines were generated by Transgenic RNAi Project (TRiP) (Perkins et al. 2015).

To compare the strength of the drivers in the different gene expression systems, *mCD8-GFP* driven by a corresponding driver was visualized in the ovary or brain (Supplementary Fig. 1). *GR1-Gal4* exhibited weaker expression in stretch follicle cells compared to *tj-Gal4* (Supplementary Fig. 1a and b). No detectable difference in GFP fluorescence intensity or expression pattern was observed between *tub-QF2-* and *tub-lexA-*driven expressions in stretch follicle cells (Supplementary Fig. 1c and d), and the level was comparable to *tj-Gal4*, although *tj-Gal4* showed higher expression in more posterior follicle cells. Similarly, no obvious difference in GFP fluorescence or expression pattern was detected between *repo-Gal4* and *repo-QF2*, while *repo-lexA* drove higher expression in astrocyte-like glia within neuropil (Supplementary Fig. 1e to g). *tub-lexA*-driven expression expressed more highly in neurons and the neuropil (Supplementary Fig. 1h), which was dramatically different from the glia-specific expression pattern driven by *repo*.

### Immunostaining

For ovary dissection, starting at 3 d post eclosion (dpe), female flies were conditioned with supplemental yeast paste for 48 h, with flies switched to fresh yeast paste daily. The 5 dpe female flies were anesthetized using CO_2_, and ovaries were dissected in 1× phosphate-buffered saline (PBS) with 0.1% Triton-X 100 (0.1% PBT) at room temperature (RT). Dissected ovaries were fixed with 4% paraformaldehyde (PFA; Thermo Fisher Scientific, #043368.9M) for 20 min and washed 3 times in 0.1% PBT for 20 min each. The last wash was removed and 2 drops of 4′,6-diamidino-2-phenylindole (DAPI; Vector Laboratories, #H-1200) were added, and samples were stored at 4 °C overnight before mounting on slides.

For brain dissection, 7 dpe flies were first fixed with 4% PFA in 0.3% PBT for 3 h at RT and washed 4 times with 0.3% PBT within 1 h. Brains were dissected in PBS at RT. Brains were permeabilized with 0.3% PBT 1 h at RT or overnight at 4 °C. Samples were blocked in PBANG [0.3% PBT, 0.5% bovine serum albumin {BSA}, and 5% normal goat serum {NGS}] 1 h at RT or overnight at 4 °C and then incubated in primary antibody (Draper 5D14, Developmental Studies Hybridoma Bank) diluted 1:200 in PBANG for 2 nights at 4 °C. Samples were then washed in 0.3% PBT 4 times for a total of 1 h at RT, before being incubated in secondary antibody (goat anti-mouse Alexa Fluor 488, Jackson Immuno, #115-545-003) diluted 1:200 in PBANG for 2 d at 4 °C in the dark. Samples were washed with PBT twice and 1× PBS twice for a total of 1 h, followed by being transferred to Vectashield with DAPI and stored in the dark at 4 °C until mounting on slides. All the incubation and washes were done with rotation.

For whole-mount phalloidin staining, whole brains were dissected, fixed, and permeabilized as described above and were incubated at 4 °C in the dark in a solution of 1:100 rhodamine phalloidin (Invitrogen, R415) diluted in 0.3% PBT overnight. Samples were washed 3 times briefly in PBS and mounted as described above.

For TUNEL staining in the brain, the In Situ Cell Death Detection Kit, Fluorescein (Roche, Cat# 11684795910) was used. Whole brains were dissected, fixed, and permeabilized as described above. Tissue was further permeabilized in freshly made 0.1% sodium citrate in 0.3% PBT for 30 min at 65 °C. Samples were then incubated in 30 µL TUNEL reaction mixture (27 µL label solution and 3 µL enzyme solution) for 3 h at 37 °C. Samples were washed 3 times in PBS while rotating for a total of 30 min. Samples were then placed in Vectashield with DAPI overnight and mounted on slides.

### Quantitative real-time polymerase chain reaction and reverse transcription-polymerase chain reaction

Flies were aged to 7 to 9 dpe. Flies were then placed in cryovials and snap frozen in liquid nitrogen in groups of 30 (15 males and 15 females). Flies were stored at −80 °C until ready for use or processed immediately. Frozen flies were decapitated by vortexing for 10 s, placing back in liquid nitrogen, and then repeating the process once more. Heads were separated from bodies rapidly using a paintbrush, over dry ice. For brain and ovary dissection, dissection chambers were cleaned with RNAase Away reagent. Flies were anesthetized on CO_2_ pad and dissected in cold nuclease-free PBS. Samples were transferred to clean, nuclease-free Eppendorf tubes using a P20 pipette. Tubes were snap frozen in liquid nitrogen. RNA was extracted using the Quick-RNA Microprep kit (ZYMO Research, R2030). RNA was stored at −80 °C until cDNA synthesis.

The Maxima First Strand cDNA Synthesis Kit for quantitative real-time polymerase chain reaction (qRT-PCR) (Thermo Scientific, K1671) was used to produce cDNA. Kit components and samples were thawed on ice, and RNA concentration was determined using a NanoDrop. Sterile RNase-free tubes on ice were used for reactions. To each tube, the following were added: 1 µL of 10× dsDNase buffer, 1 µL of dsDNase, 0.1 µg of total RNA, and nuclease-free water to reach 10 µL. Reactions were gently mixed and briefly centrifuged on a table-top centrifuge. Reactions were then incubated at 37 °C for 2 min and then chilled on ice. The following components were then added to each tube: 4 µL of 5× Reaction Mix, 2 µL of Maxima Enzyme Mix, and 4 µL of nuclease-free water. Reactions were gently mixed and briefly centrifuged on a table-top centrifuge. Reactions were then incubated for 10 min at 25 °C, followed by 15 min at 50 °C, and then 5 min at 85 °C. The reaction products were then immediately used for reverse transcription-polymerase chain reaction (RT-PCR) and qRT-PCR.

The LongAmplification *Taq* polymerase kit (NEB #M0323S) was used to perform RT-PCR. In nuclease-free tubes on ice, samples were prepared for each genotype using 12 µL DI H_2_O, 4 µL 5× LongAmp *Taq* buffer, 0.6-µL 10 mM dNTP, 1 µL template DNA, 0.8 µL LongAmp *Taq* enzyme, 0.8 µL 10 µM primer forward, and 0.8 µL 10 µM primer reverse. In the BioRad T100 thermocycler, samples used the PCR program of 30 s at 94 °C, 15 s at 94 °C, 30 s at 61.5 °C, and 50 s/kb length of amplicon at 68 °C for 30 cycles, followed by 10 min at 68 °C and storage at 4 °C. Samples were either immediately used or stored at −20 °C. Primer information is listed in Table 1. qRT-PCR primers were designed using FlyPrimerBank (Hu et al. 2013).

**Table 1.** Primer sequences for qRT-PCR and RT-PCR.

Experiment	Primer	Sequence 5′-3′
qRT-PCR	*rpl32* forward	ATGCTAAGCTGTCGCACAAATG
	*rpl32* reverse	GTTCGATCCGTAACCGATGT
	*drpr* forward	GCAGGGTGGGTAGCTATTCG
	*drpr* reverse	ATTCATCGGGATCCTTGTAGC
RT-PCR	E7 reverse (RT-PCR)	GGAATGCAAGGAACGGTGCCC
	E4 forward (RT-PCR)	CATGGTTACGGAGGACCCGCC
PCR	5′ gRNA forward	CCAAAGATCTGCACGTAGTGTC
	3′ gRNA reverse	TGCAAGACAAATGCTACGCTAC

The 2× UltraSYBR Mixture (High ROX) (CWBIO, 2602) was used to perform qRT-PCR. In nuclease-free tubes on ice, master mixes were prepared for each primer with final concentrations of primers and cDNA templates as 200 nM and 4 ng/µL, respectively, and 1× UltraSYBR Mixture (High ROX). In a 384-well plate, 10 µL of mixture was loaded into each well. The plate was sealed with a clear plate cover and then briefly centrifuged. Samples were then analyzed in an ABI 7900HT real-time PCR machine.

### Microscopy and quantification

For PNC nuclei Assay, mature eggs were identified as stage 14 (S14) egg chambers that had developed dorsal appendages but retained their follicle cell layer. PNC nuclei were numerically quantified on an Olympus BX60 microscope. For each sample, the total number of nurse cell nuclei was divided by the number of S14 egg chambers scored. Negative binomial regression or Poisson regression, with or without zero inflation, was used for statistical analysis using R.

Brains mounted on slides were imaged by confocal microscopy (Nikon AX). For neurodegeneration analysis, brains stained with phalloidin were imaged with a 20× objective with a numerical aperture of 0.8 and a working distance of 0.8 mm. Z-stacks were acquired at 1 µm using the Nikon NIS-Elements software with a resolution of 1024 × 1024 pixels. Images were processed through Fiji and Webknossos as described (Liu et al. 2024). Images were converted to RGB Z-stacks in Fiji and then imported to Webknossos for vacuole segmentation and 3-dimensional geometric analysis. Areas devoid of staining were identified as vacuoles using the “quick select” automatic segmentation tool. Segmentation was performed for selected layers of the Z-stack, and volume interpolation was performed to annotate the vacuole regions within the segmentation layers. 3D meshes were computed for volume interpolated vacuoles in Webknossos, and corresponding statistical.csv files containing number and volume information of vacuoles were exported for downstream analysis. The total number of vacuoles per sample and the percentage of whole-brain volume occupied by vacuoles were determined using R. Negative binomial regression or Poisson regression, with or without zero inflation, was performed for vacuole count statistical analysis using R. Two-tailed *t*-test and 1-way ANOVA were performed for vacuole volume in R.

For Drpr and TUNEL staining, brains were imaged with a 40× objective with a numerical aperture of 0.95 and a working distance of 0.21 mm. Drpr images were acquired at a resolution of 1024 × 1024 pixels and TUNEL images at 2048 × 2048 pixels. The number of TUNEL-positive cells was quantified using the cell counter plugin in Fiji. Negative binomial regression or Poisson regression, with or without zero inflation, was used for statistical analysis using R.

### CRISPR/Cas9

gRNA sequences corresponding to regions outside exons 5 and 12 in the *drpr RE* isoform (FlyBase) were identified through https://www.flyrnai.org/crispr/ (Table 2). Oligos were annealed and ligated into plasmid pCFD3:dU6 (Addgene #49410) following the protocol from https://crisprflydesign.org/ (Port et al. 2014). Plasmids expressing the 5′ and 3′ gRNAs were injected into *vas-Cas9* embryos (BDSC #51323) by BestGene Inc. Individual lines were established from progeny from the injected larvae. One line that was derived from the injected larvae was found to have a wild-type *drpr* sequence, and this was used as a control line (*ctrl1*) for future experiments. We used FlyBase (FB2025_01) to obtain the sequence (Jenkins et al. 2022).

**Table 2.** CRISPR design.

Name	Sequence
3′ gRNA	ACTTAAACTTACGTACCAGTTGG
Sense oligo	GTCGACTTAAACTTACGTACCAGT
Antisense oligo	AAACACTGGTACGTAAGTTTAAGT
5′ gRNA	AGAGAGAGAGAGTGGAGTGTAGG
Sense oligo	GTCGAGAGAGAGAGAGTGGAGTGT
Antisense oligo	AAACACACTCCACTCTCTCTCTCT

### DNA extraction

A total of 100 µL of squish buffer solution (10 µL 10 mM Tris–HCl, 1 µL 1-mM EDTA, 2.5 µL 25 mM NaCl, 5 µL 200-µg/mL proteinase K, 481.5 µL diH_2_O) was placed in a 1.5 mL Eppendorf tube along with 2 anesthetized flies of the appropriate genotype. The whole flies and squish buffer solution were homogenized using a motor pestle, and the sample was incubated at RT for 15 min. The vials were then added to a 95 °C heat block for 3 min to denature the proteinase K. The supernatant containing DNA was retained after centrifuging at 14,000 × *g* for 10 min. The DNA to be used in PCR experiments was either used immediately or stored at −20 °C.

### Polymerase chain reaction

Polymerase chain reaction (PCR) was performed to verify whether the CRISPR/Cas9-manipulated flies contained deletions using the primers in Table 1. The LongAmplification *Taq* polymerase kit (NEB #M0323S) was used to perform PCR using a program of 15 s at 94 °C, 30 s at 61.5 °C, and 3 min and 45 s (50 s/kb length of amplicon) at 68 °C for 30 cycles.

### Western blotting

Flies at 7 dpe were anesthetized using CO_2_, and their heads were decapitated and snap frozen. Frozen fly heads (15 males and 15 females) were homogenized in 100 µL of ice-cold lysis buffer. Lysates were incubated on ice for 1 h with vortexing every 20 min. Samples were then centrifuged at 14,000 × *g* for 15 min at 4 °C. Supernatants were collected and either used immediately or stored at −20 °C for later use.

Protein concentration was measured using the Micro BCA Protein Assay Kit (Thermo Fisher Scientific, #23235). A total of 50 µg of head protein extracts and 10 µL of protein standard (BioRad, 1610374EDU) were mixed with 1× NuPAGE^TM^ LDS Sample Buffer (Thermo Fisher Scientific, #NP0007) and incubated at 37 °C for 10 min. A total of 15 µL of each sample was loaded onto a 4% to 12% NuPAGE™ Bis-Tris Mini Protein Gels (Invitrogen, #NP0322BOX). The gel was run in MOPS buffer at 150 V for 1 h and then transferred onto a methanol-activated PVDF membrane (Invitrogen, #PB9220) using the Power Blotter System (Invitrogen, #PB0012) at 25 V and 1.5 mA for 9 min. Membranes were blocked with 5% nonfat milk in TBST (150 mM NaCl, 20 mM Tris, pH 7.5, 0.1% Tween-20) for 1 h at RT, followed by incubation with primary antibody overnight at 4 °C. Membranes were washed 3 × 10 min in TBST at RT and then incubated with secondary antibody at RT for 1 h. Primary antibodies used were mouse anti-Draper (1:500; DSHB, 5D14) and mouse anti-Lamin (1:500; DSHB, ADL84.12). The secondary antibody was goat anti-mouse HRP-conjugated IgG (1:10,000; Jackson Immuno Research Labs, #115-036-068). Proteins were detected with ECL substrate (Thermo Scientific, #34577) and imaged with a Sapphire Biomolecular Imager (Azure Biosystems).

## Results

### Multiple *drpr^RNAi^* lines consistently abolish Drpr immunostaining

To systematically compare the knockdown efficiency of different *drpr^RNAi^* lines, we examined commonly used UAS/GAL4 and QUAS/QF2 systems. Specifically, we tested 2 *UAS-drpr^RNAi^* lines and 1 *QUAS-drpr^RNAi^* line. The UAS lines differ in their RNAi constructs: one expresses a LH dsRNA (MacDonald et al. 2006), while the other expresses a SH RNA in the VALIUM20 vector (Perkins et al. 2015). The *QUAS-drpr^RNAi^* line was generated using the *pQUAS-WALIUM20* vector (Perkins et al. 2015).

**Fig. 1.**
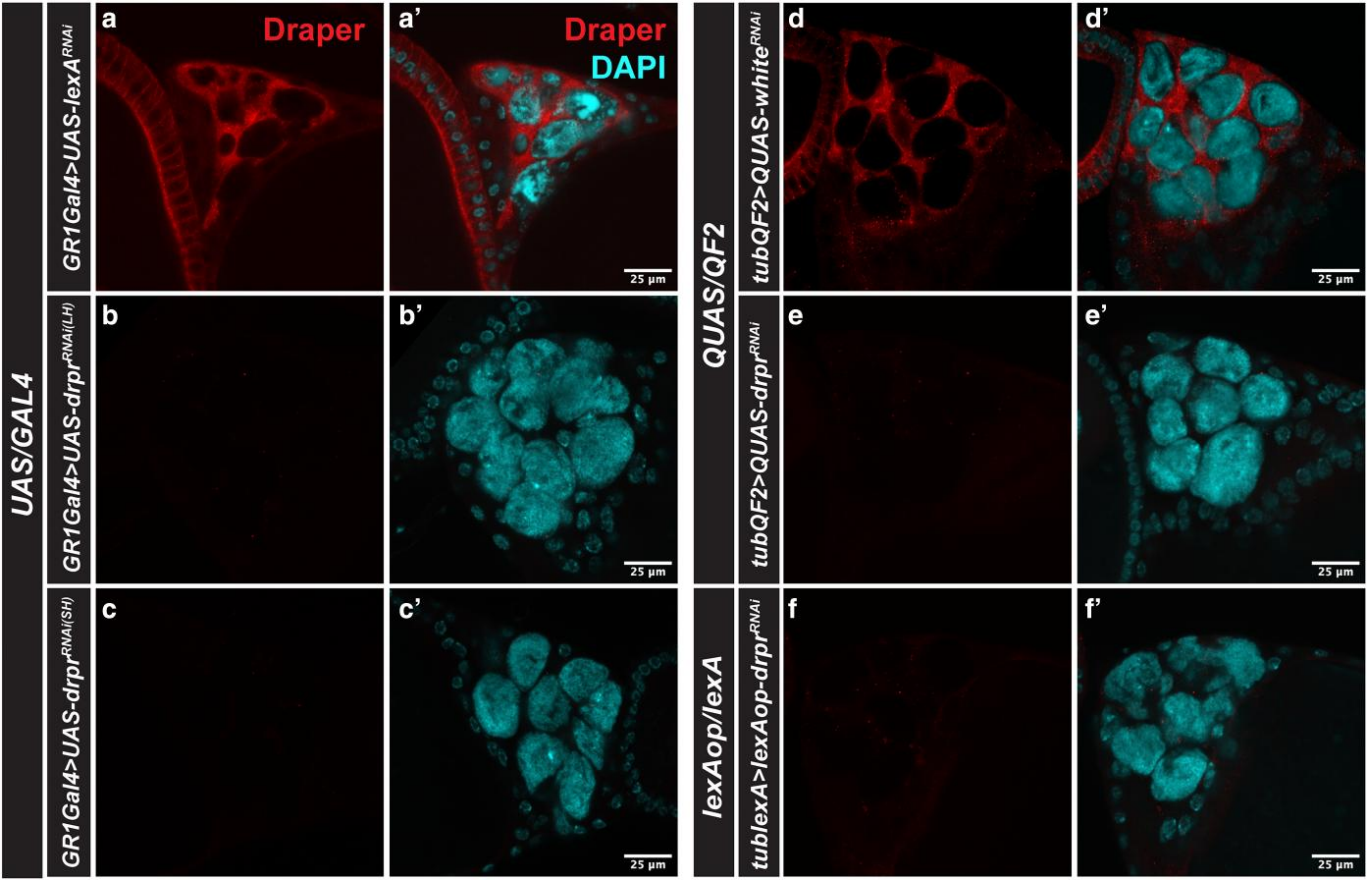
Draper expression in the ovary. The anterior portions of stage 12 egg chambers stained with Draper (Drpr, red) and DAPI (cyan) in flies expressing *drpr^RNAi^* or indicated controls using *UAS/GAL4* (a to c’), *QUAS/QF2* (d to e’), and *lexAop/lexA* (f to f’) systems. Scale bar, 25 µm.

We first performed Drpr immunostaining in 5 to 7 dpe fly ovaries and brains to assess knockdown efficiency at the protein level. We used *GR1-Gal4* to drive the expression of *UAS-drpr^RNAi^* or *UAS-lexA^RNAi^* (control) in somatic follicle cells. We focused on stretch follicle cells in stage 12 egg chambers, which normally show strong Drpr expression (Fig. 1a to a’). Both LH and SH constructs showed an absence of Drpr staining in stretch follicle cells, with no discernible difference between the 2 lines (Fig. 1a to c’). Due to the unavailability of a *GR1-QF2* driver, we instead used the *tubulin-QF2 (tub-QF2)* driver to express *QUAS-drpr^RNAi^* or *QUAS-w^RNAi^* (control) ubiquitously (Fig. 1d to f’). In *tub-QF2 > QUAS-drpr^RNAi^* and *tub-lexA > lexAop-drpr^RNAi^* ovaries, Drpr staining was also absent, although some residual Drpr-positive puncta were detected (Fig. 1e and f’), suggesting slightly less efficient knockdown compared to *GR1-Gal4 > UAS-drpr^RNAi^* (Fig. 1c).

**Fig. 2.**
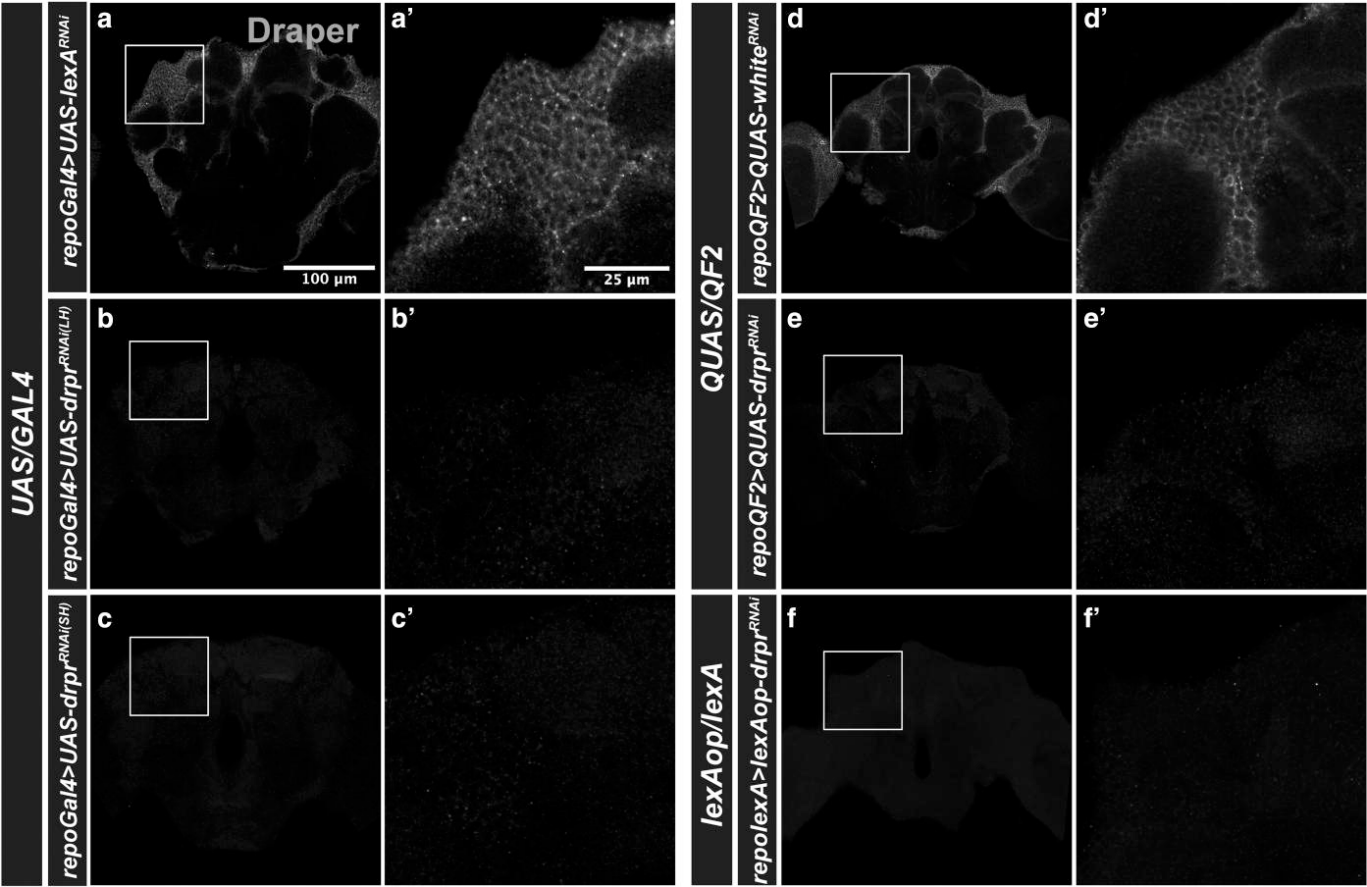
Drpr expression in the brain. Brains stained with Drpr in flies expressing *drpr^RNAi^* or indicated controls using *UAS/GAL4* (a to c’), *QUAS/QF2* (d to e’), and *lexAop/lexA* (f to f’) systems. Main panel scale bar, 100 µm; zoom-in panels, 25 µm.

We next assessed knockdown efficiency in the adult brain by driving *drpr^RNAi^* expression compared to controls in glial cells using the *repo-Gal4* and *repo-QF2* drivers. Consistent with results in the ovary, both LH and SH *UAS-drpr^RNAi^* constructs driven by *repo-Gal4* eliminated detectable Drpr signal in glia (Fig. 2a to c’). Similarly, expression of *QUAS-drpr^RNAi^* under *repo-QF2* and *lexAop-drpr^RNAi^* under *repo-lexA* also led to efficient knockdown of Drpr (Fig. 2d to f’), with staining levels comparable to those seen in *repo-Gal4 > UAS-drpr^RNAi^* brains.

### *drprRNAi* lines exhibit variable mRNA levels in whole-tissue quantification

Although these *drpr* RNAi lines reliably eliminated detectable Drpr protein in both ovarian follicle cells and glia by immunostaining, immunofluorescence does not provide precise measurements of knockdown efficiency. To obtain a quantitative assessment, we performed qRT-PCR on tissues collected from 7 dpe flies to measure drpr mRNA levels. Primer pairs were designed using the FlyPrimerBank to target a shared region that is common to all known drpr splice variants, enabling quantification of total drpr transcript levels (Hu et al. 2013).

**Fig. 3.**
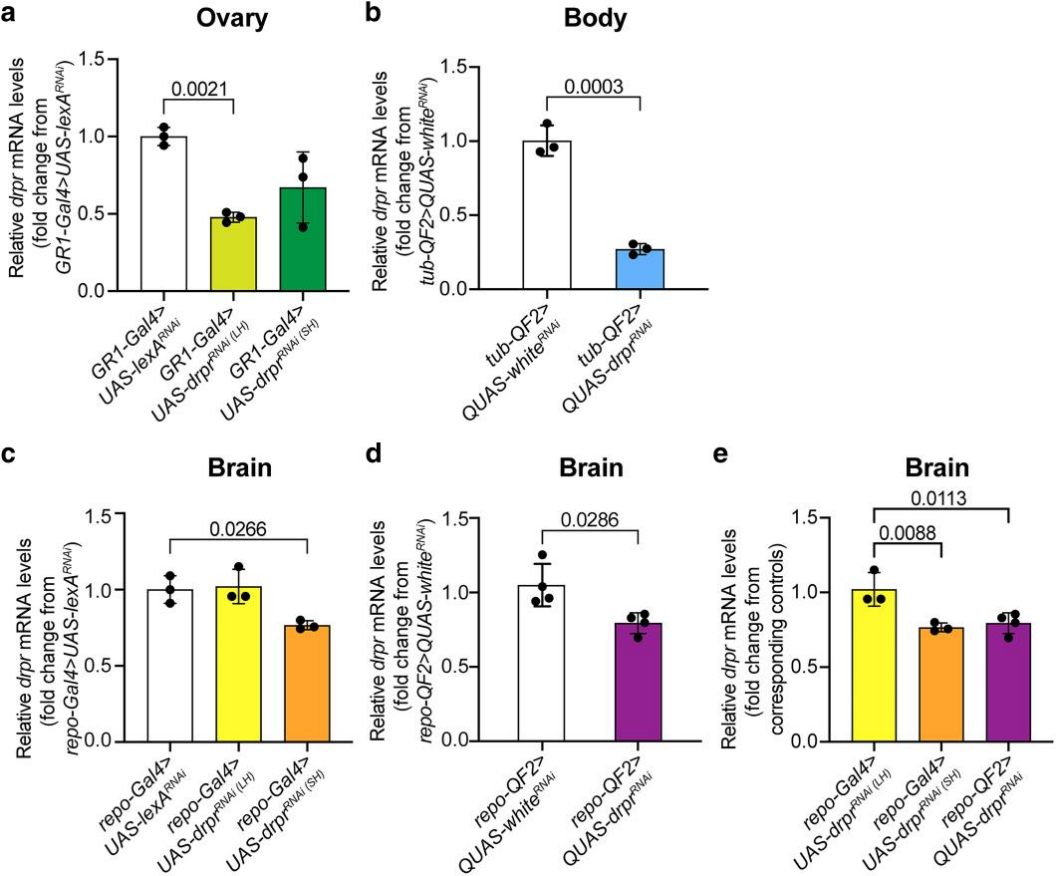
*Drpr* mRNA levels in whole tissue across genotypes measured by qPCR. a) Log fold change in *drpr* expression in ovaries of *UAS/GAL4*-driven RNAi flies, normalized to *GR1-Gal4 > UAS-lexA^RNAi^* controls. b) Log fold change in *drpr* expression in whole bodies of *QUAS/QF2*-driven RNAi flies, normalized to *tub-QF2 > QUAS-white^RNAi^* controls. c) Log fold change in *drpr* expression in brains of *UAS/GAL4*-driven RNAi flies, normalized to *repo-Gal4 > UAS-lexA^RNAi^* controls. d) Log fold change in *drpr* expression in brains of *QUAS/QF2*-driven RNAi flies, normalized to *repo-QF2 > QUAS-white^RNAi^* controls. e) Comparison of *drpr* expression levels in brains across *UAS/GAL4* and *QUAS/QF2* systems normalized to their respective controls. Each dot represents 1 replicate; *n* = 30 flies per genotype. Data are mean ± SD. Statistical significance was assessed using nonparametric Student’s *t*-test for 2-group comparisons and 1-way ANOVA with Dunnett's posttest for multiple comparisons (Prism software). *P* < 0.05 was considered significant.

In the ovary, the LH *UAS-drpr^RNAi^* line significantly reduced *drpr* mRNA levels compared to controls (Fig. 3a). The LH construct exhibited a stronger, though not statistically significant, knockdown effect, reducing mRNA levels by approximately 50%, compared to ∼30% in the SH line (Fig. 3a). The incomplete mRNA knockdown could reflect expression of *drpr* in the germline or subsets of follicle cells that do not express *GR1-Gal4*. For the assessment of ubiquitous *drpr* knockdown using the *tub-QF2 > QUAS-drpr^RNAi^* line, whole-body extracts were analyzed. These flies showed a significant ∼75% reduction in *drpr* mRNA levels relative to controls (Fig. 3b).

In dissected adult brains, *repo-Gal4 > UAS-drpr^RNAi(SH)^* significantly reduced drpr transcript levels, while the LH construct did not show a significant effect (Fig. 3c). Similarly, *repo-QF2 > QUAS-drpr^RNAi^* flies also displayed a significant reduction in brain *drpr* mRNA levels compared to controls (Fig. 3d). Moreover, the knockdown efficiency of *repo-QF2 > QUAS-drpr^RNAi^* was comparable to that of *repo-Gal4 > UAS-drpr^RNAi(SH)^* (Fig. 3e), suggesting that the SH construct is more effective in glial cells. Although *repo-Gal4 > UAS-drpr^RNAi(LH)^* did not significantly reduce *drpr* mRNA level in the brain, Drpr immunostaining is absent (Fig. 2b to b’). This suggests that the LH construct may interfere with Drpr translation (Mohr et al. 2014). The residual expression of *drpr* in the brains of *repo > drpr^RNAi^* flies, using both the UAS/GAL4 and QUAS/QF2 systems, may reflect *drpr* expression in other cell types not targeted by the *repo* driver, including neurons and hemocytes (Li et al. 2022).

Together, these results highlight tissue-specific differences in RNAi efficiency, which may reflect variations in driver strength, RNAi construct design, or differential processing of hairpin RNAs in distinct cellular contexts.

### Strong PNC nuclei phenotypes in the ovary from *drpr^RNAi^* lines

**Fig. 4.**
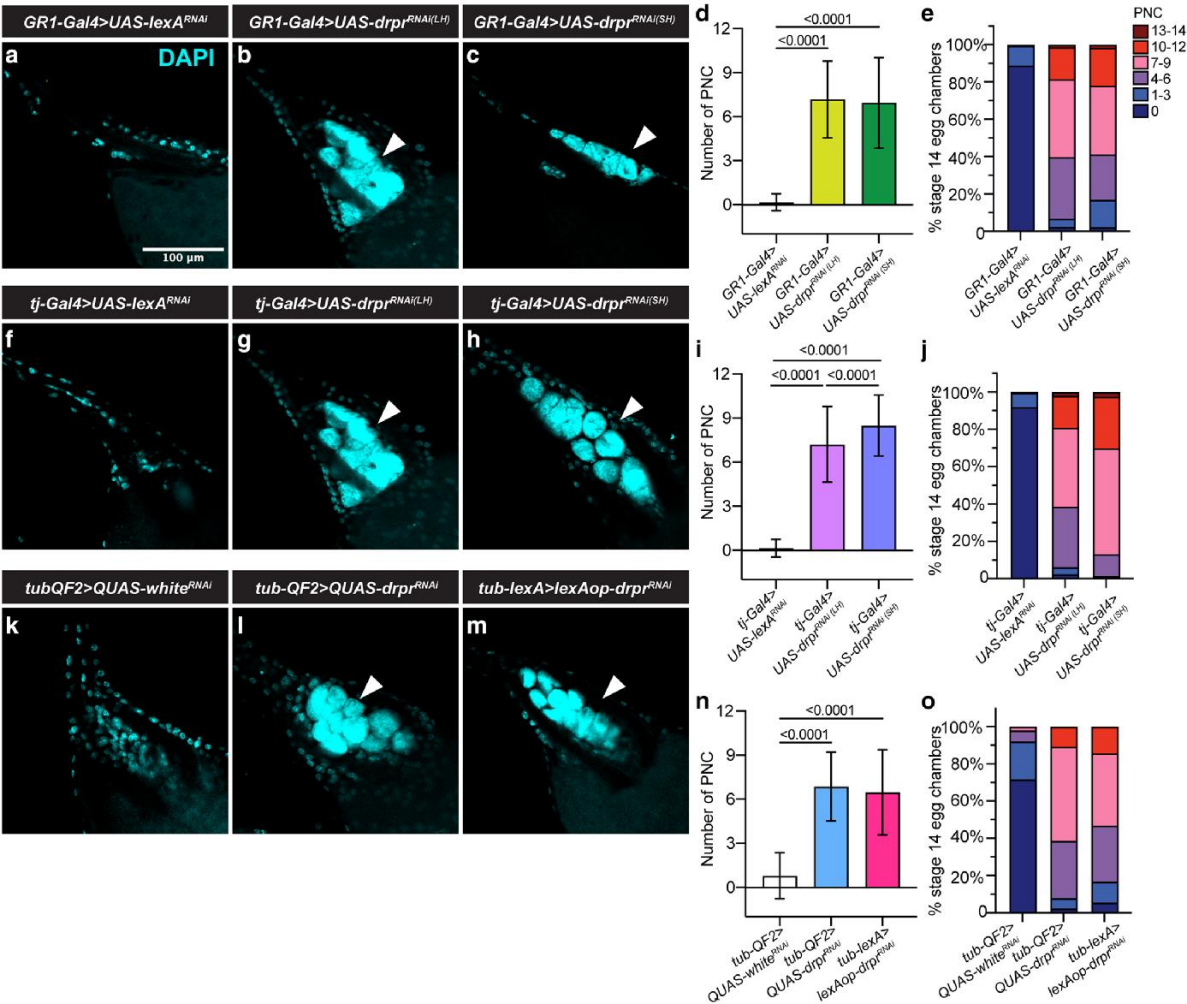
PNC nuclei in the ovary following *drpr* knockdown. a to c, f to h, and k to m) Representative images of mature stage 14 (S14) egg chambers stained with DAPI showing PNC nuclei in indicated genotypes. PNC nuclei are indicated by white arrowheads. Smaller nuclei correspond to follicle cells. Scale bar, 100 µm. d, i, and n) Quantification of PNC nuclei per egg chamber. *GR1 > lexA^RNAi^*, *n* = 224 egg chambers; *GR1 > drpr^RNAi(LH)^*, *n* = 772; *GR1 > drpr^RNAi(SH)^*, *n* = 231. *tj > lexA^RNAi^*, *n* = 410; *tj > drpr^RNAi(LH)^*, *n* = 674; *tj > drpr^RNAi(SH)^*, *n* = 399. *tub-QF2 > QUAS-white^RNAi^*, *n* = 363; *tub-QF2 > QUAS-drpr^RNAi^*, *n* = 166; *tub-lexA > lexAop-drpr^RNAi^*, *n* = 384. Statistical significance was assessed using zero-inflated negative binomial model (R software). *P* < 0.05 was considered significant. e, j, and o) Alternative quantification of data presented in (d, i, and n). The number of persisting nuclei per stage 14 egg chamber was categorized into bins of 0, 1 to 3, 4 to 6, 7 to 9, 10 to 12, and 13 to 14 PNC, and the percentage of stage 14 egg chambers in each bin was calculated.

To determine whether varying degrees of *drpr* knockdown produce differential ovarian phenotypes, we quantified the number of PNC nuclei in mature stage 14 (S14) egg chambers (Timmons et al. 2016). In *GR1-Gal4 > UAS-drpr^RNAi^* flies, both LH and SH lines exhibited a significant increase in PNC nuclei per S14 egg chamber compared to controls, with no significant difference observed between the 2 RNAi lines (Fig. 4a to e).

We also assessed *drpr* knockdown using a second driver, *traffic jam (tj)-Gal4*, which also targets follicle cells. In *tj-Gal4 > UAS-drpr^RNAi^* flies, both LH and SH lines also showed significantly elevated PNC numbers compared to controls (Fig. 4f to j). Moreover, the SH line exhibited a higher number of PNC nuclei than the LH line (Fig. 4i).

Furthermore, *tub-QF2 > QUAS-drpr^RNAi^* flies also displayed a significant increase in PNC nuclei number compared to controls (Fig. 4k, l, n, and o), confirming the effective *drpr* knockdown in the QF2/QUAS system, consistent with the original characterization of this line (Perkins et al. 2015). Similarly, *tub-lexA > lexAop-drpr^RNAi^* flies exhibited significantly more PNC nuclei than *tub-QF2 > QUAS-white^RNAi^* controls, though slightly fewer, albeit not significantly, than *tub-QF2 > QUAS-drpr^RNAi^* flies (Fig. 4m to o).

### SH *drpr^RNAi^* shows more persisting cell nuclei in the brain

**Fig. 5.**
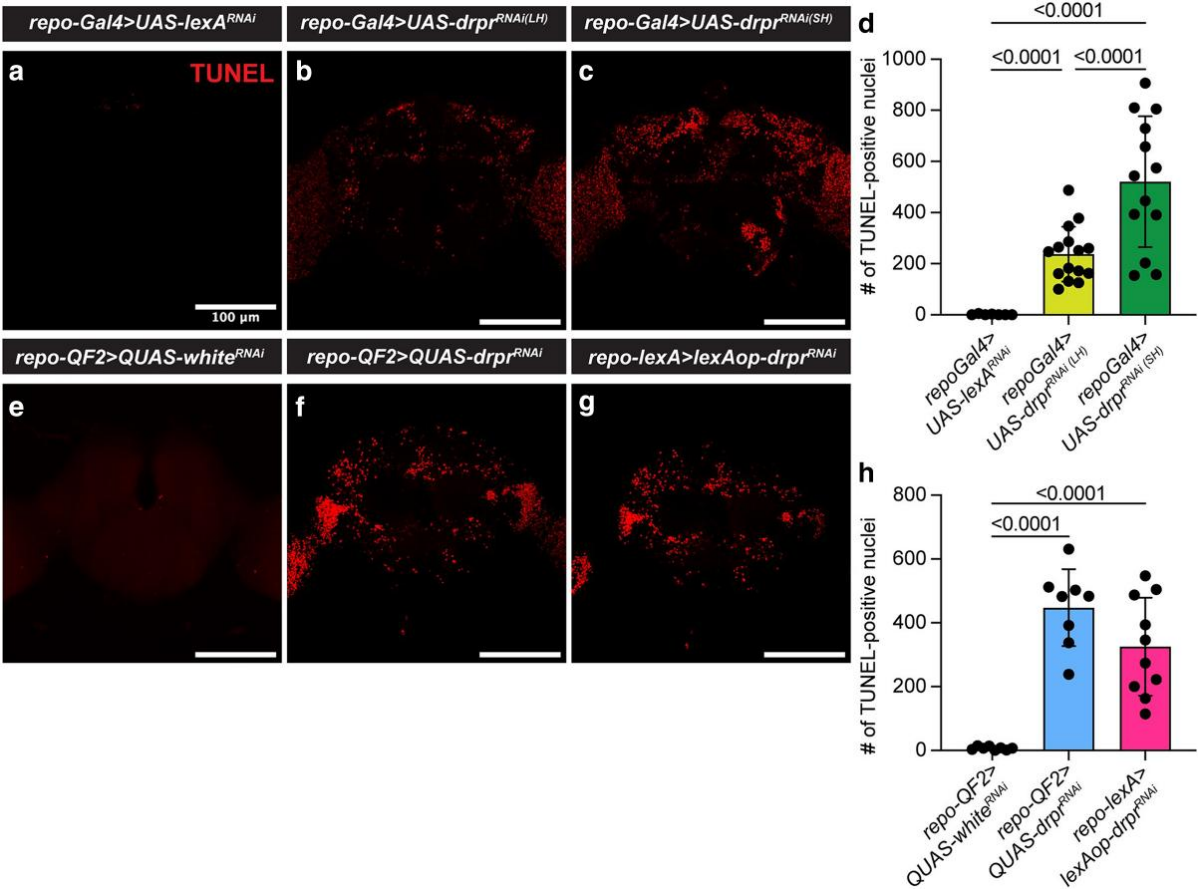
Persisting dead cells in the brain. a to c and e to g) Representative projections (30 µm thick) of anterior brain regions from 7-d-old adult flies showing TUNEL-positive cell corpses (red). Scale bar, 100 µm. d and h) Quantification of TUNEL-positive cells in the central brain across genotypes. Each point represents 1 brain. *repo > lexA^RNAi^*, *n* = 7 brains; *repo > drpr^RNAi(LH)^*, *n* = 15; *repo > drpr^RNAi(SH)^*, *n* = 13. *repo-QF2 > QUAS-white^RNAi^*, *n* = 8; *repo-QF2 > QUAS-drpr^RNAi^*, *n* = 8; *repo-lexA > lexAop-drpr^RNAi^*, *n* = 10. Data are mean ± SD. Statistical significance was assessed using zero-inflated negative binomial model (R software). *P* < 0.05 was considered significant.

Knocking down *drpr* in glia leads to the persistence of dead cells (TUNEL positive), both neurons and glia, in the adult brain (Etchegaray et al. 2016). Using the TUNEL assay, we quantified dead cells in the central brains of 7-d-old flies. In the UAS/GAL4 system, the LH line showed a significant increase in TUNEL-positive signals compared with controls, and the SH line displayed even more TUNEL-positive cells than the LH line (Fig. 5a to d), indicating a stronger knockdown effect.

Consistent with these results, *repo-QF2 > QUAS-drpr^RNAi^* flies also exhibited a significant increase in TUNEL-positive cells compared to controls (Fig. 5e to h), confirming the effective *drpr* knockdown in the QF2/QUAS system. Similarly, *repo-lexA > lexAop-drpr^RNAi^* flies showed significantly more dead cells than *repo-QF2 > QUAS-white^RNAi^* controls, although the number was slightly lower but not significantly than that observed in *repo-QF2 > QUAS-drpr^RNAi^* flies (Fig. 5h).

### SH *drpr^RNAi^* shows more severe neurodegeneration phenotypes

**Fig. 6.**
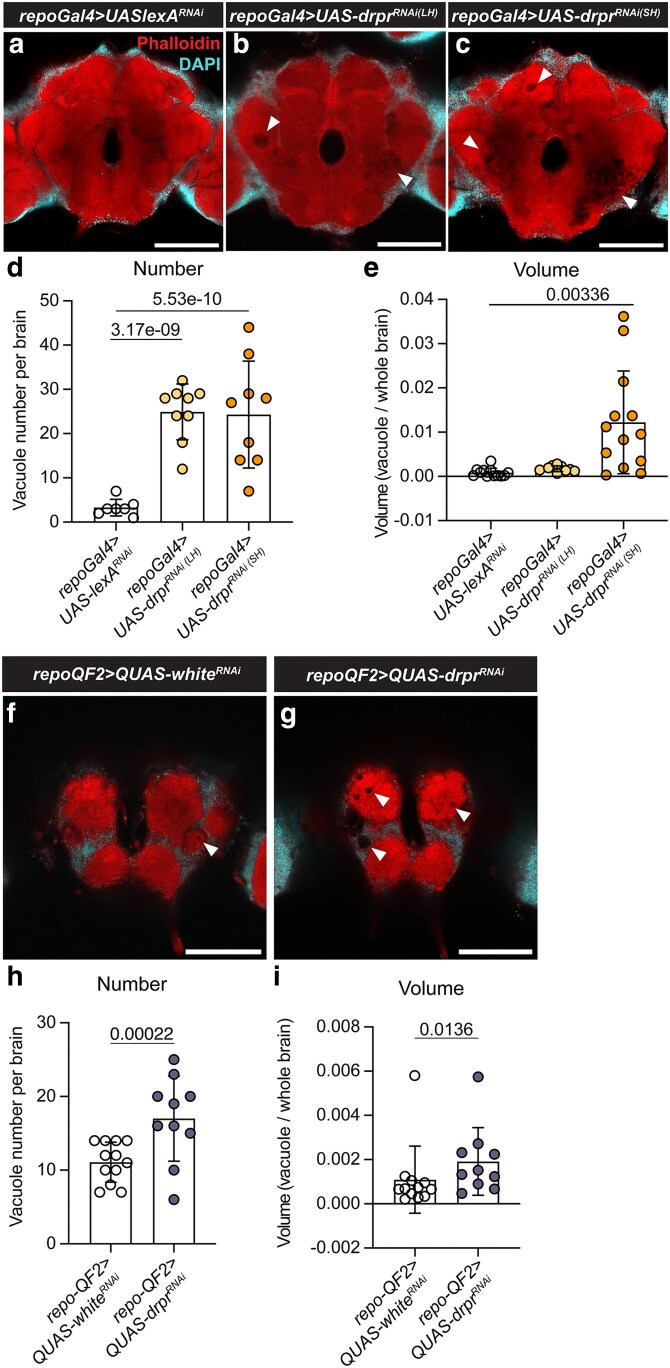
Neurodegeneration in aged brains. a to c) Representative images of 40-d-old brains showing periesophageal neuropils from *UAS/GAL4*-driven RNAi flies stained with phalloidin (red) and DAPI (cyan). Vacuoles are indicated by white arrowheads as black holes. Scale bar, 100 µm. Brain region nomenclature follows (Ito et al. 2014). d and e) Quantification of vacuole number (d) and volume (e) in 40-d-old brains of *UAS/GAL4*-driven RNAi flies. Each data point represents 1 brain; *repo > lexA^RNAi^*, *n* = 7; *repo > drpr^RNAi(LH)^*, *n* = 9; *repo > drpr^RNAi(SH)^*, *n* = 9. Negative binomial model was performed for vacuole number. One-way ANOVA with Dunnett's posttest was performed for vacuole volume (R software). *P* < 0.05 was considered significant. f and g) Representative images of 40-d-old brains showing anterior regions, including antennal lobes, from Q*UAS/QF2*-driven RNAi flies, stained as in (a to c). Vacuoles are indicated by arrowheads. Scale bar, 100 µm. h and i) Quantification of vacuole number (h) and volume (i) in 40-d-old brains of Q*UAS/QF2*-driven RNAi flies. Each data point represents 1 brain; (h) *repo-QF2 > QUAS-white^RNAi^*, *n* = 12; *repo-QF2 > QUAS-drpr^RNAi^*, *n* = 10. Statistical analysis as in (d and e).

Neuronal cell death is often accompanied by the formation of neurodegenerative vacuoles (Coombe and Heisenberg 1986; Buchanan and Benzer 1993; Kretzschmar et al. 1997; Wittmann et al. 2001). To assess neurodegenerative phenotypes associated with *drpr* knockdown, we analyzed vacuole formation in the whole-mount brains across all RNAi lines. Vacuoles are defined here as areas devoid of nuclei and F-actin staining. In *repo-Gal4 > UAS-drpr^RNAi^* flies, vacuoles were detected throughout the brain, extending from the antennal lobes at the anterior to deeper central brain regions (Fig. 6a to c). Both LH and SH RNAi lines exhibited significantly increased vacuole numbers compared to controls (Fig. 6d). However, only the SH line showed a significant increase in total vacuole volume (Fig. 6e), suggesting that vacuole volume may be a more stringent metric for detecting neurodegeneration. These findings also indicate that the SH line induces a stronger neurodegenerative effect. In *repo-QF2 > QUAS-drpr^RNAi^* flies, vacuoles were primarily localized near the antennal lobes at the anterior end (Fig. 6f and g). These flies displayed significant increases in both vacuole number and total vacuole volume compared to controls (Fig. 6h, i). However, the number and volume of vacuoles in aged flies driven by the QUAS/QF2 system were smaller than those driven by the UAS/GAL4 system, suggesting that UAS/GAL4 produces stronger neurodegenerative phenotypes. Notably, the spatial distribution of vacuoles differed between the GAL4/UAS and QF2/QUAS systems, highlighting potential differences in driver strength, spatial expression patterns, or RNAi efficacy between these systems. This observation may warrant further investigation.

### Generation of a new *drpr* allele via CRISPR/Cas9

**Fig. 7.**
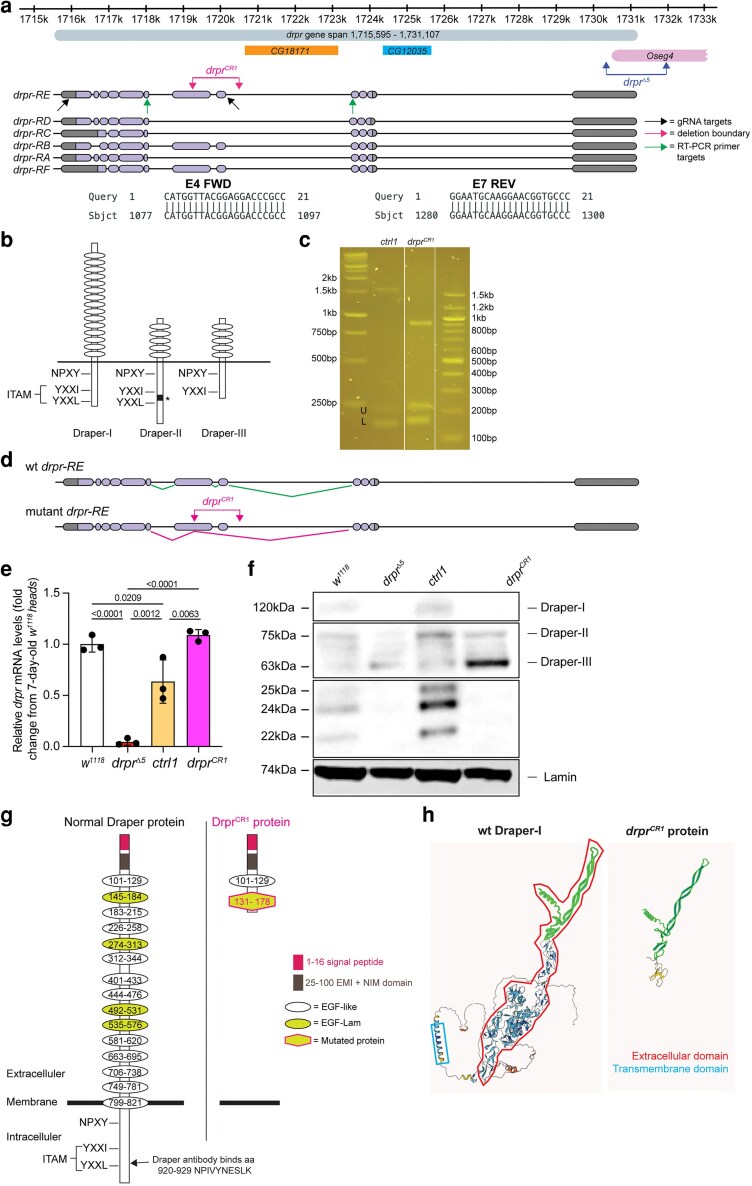
Generation and characterization of a new *drpr* allele. a) Schematic of the *drpr* gene. The gene spans 15,513 nucleotides (1,715,595 to 1,731,107) and produces 6 mRNA transcripts composed of different combinations of the 12 exons. Transcription occurs from right to left relative to the chromosome. The full gene span, including introns and exons, is shown in light gray. Coding regions of mRNAs are shown in purple and noncoding regions in gray. Neighboring genes *CG18171* and *CG12035* are indicated. Black arrows mark the CRISPR/Cas9 gRNA targets, magenta arrows mark the deletion boundaries, and green arrows mark the RT-PCR primer sites used for transcript verification. Blue arrows indicate the deletion site of the commonly used drpr^Δ5^ allele, which extends from exon 1 into the adjacent Oseg4 gene. b) Drpr isoform schematic adapted from Logan et al. (2012). Ovals represent extracellular EGF-like motifs. NPXY denotes the predicted Ced-6 binding site. Drpr-I contains an ITAM (YXXI-X11-YXXL) that binds Shark. Drpr-II contains a unique insertion (asterisk) within the ITAM and Drpr-III lacks an ITAM due to a frameshift and premature stop codon. c) RT-PCR analysis of *ctrl1* and *drpr^CR1^* flies. Agarose gel showing PCR products spanning the mutation region. Three band sizes were detected in both genotypes, with 1 fragment differing. Bands slightly larger than 200 bp (U: *drpr-RD*) and slightly smaller than 200 bp (L: *drpr-RA/RC*) were also observed. Full gel can be found in Supplementary Fig. 2a. d) Schematic of *drpr-RE* splicing. Top: wild-type splicing from exon 4 → exon 5 → exon 6 → exon 7. Bottom: proposed splicing in *drpr^CR1^*, in which exon 4 bypasses the deleted region of exons 5 to 6, splicing directly to the next nucleotide after the deletion. Wild-type transcripts are shown in green and mutant transcripts in magenta. e) Log fold change of *drpr* expression in heads of *w^1118^*, *drpr^Δ5^*, *ctrl1*, and *drpr^CR1^*, normalized to *w^1118^*. Each dot represents 1 replicate (*n* = 30 flies per genotype). Data are mean ± SD. Statistical significance was assessed by nonparametric Student’s *t*-test for 2-group comparisons and 1-way ANOVA with Dunnett's posttest for multiple comparisons (Prism software). *P* < 0.05 was considered significant. f) Western blot of Drpr in head lysates from *w^1118^*, *drpr^Δ5^*, *ctrl1*, and *drpr^CR1^* with Lamin as a loading control. Full gel can be found in Supplementary Fig. 2b. g) Schematic of normal and mutant Draper protein structure. h) AlphaFold-predicted structures of wild-type and mutated Drpr protein. Green highlights denote regions unaffected by the mutation. The cyan box marks the transmembrane domain and red outlines the extracellular domain. The frameshift mutation introduces a premature stop codon, producing a truncated protein. Green regions show portions of the wild-type protein predicted to be present in both wild-type Drpr-I and mutant Drpr-I.

Most analysis of *drpr* has been conducted with the *drpr^Δ5^* allele, which deletes the promoter and part of the 5′ UTR, along with a portion of the neighboring gene *Oseg4*, which is required for anterograde transport in distal cilia (Fig. 7a) (Freeman et al. 2003). This allele is frequently referred to as a null allele; however, since the *drpr* coding region is intact, we wished to generate a deletion that removed as much of the coding region as possible. Two genes, *CG18171* and *CG12035*, are nested within introns of *drpr*; thus, we designed gRNAs to target outside exons 5 and 12 (of splice form RE), which would not affect those genes (Fig. 7a) (FB2025_01) (Jenkins et al. 2022). Plasmids encoding these gRNAs were injected into Cas9-expressing embryos, and progeny were analyzed by phenotype, PCR, and Western blot.

One resulting line, hereafter referred to as *drpr^CR1^*, was found to carry a 1,107-bp deletion that deletes all of exon 5 and approximately half of exon 6 (Fig. 7a). The wild-type *drpr* gene undergoes alternative splicing and encodes 3 major protein isoforms—Drpr-I, Drpr-II, and Drpr-III (Fig. 7a and b) (Freeman et al. 2003; Logan et al. 2012). Drpr-I (encoded by splice form RB in FlyBase) contains a large extracellular domain and intracellular signaling motifs and functions as a phagocytic receptor. Drpr-II (splice form RA) and Drpr-III (splice form RC) lack most of the extracellular domain and differ in their cytoplasmic signaling motifs, conferring distinct downstream signaling capabilities. Drpr-II has been determined to inhibit the function of Drpr-I (Logan et al. 2012), while the action of Drpr-III remains unclear. Additional isoforms have also been identified (FB2025_01) (Jenkins et al. 2022) (Fig. 7a), further adding to the complexity.

To determine how *drpr^CR1^* affected splicing, we conducted RT-PCR with primers corresponding to exons 4 and 7 (Fig. 7a). In control (*ctrl1*) flies, we detected 3 bands corresponding to the predicted *drpr* transcripts (*RB/RE*/*RF*), *drpr-RD* and *drpr-RA/RC* (Fig. 7c). In contrast, *drpr^CR1^* flies lacked the full-length *drpr* band and produced a new band of ∼900 bp (Fig. 7c). Sequencing of the new band revealed that exon 4 was spliced to the middle of exon 6 (Fig. 7d), creating a frameshift after 130 amino acids, which corresponds to the extracellular region. Moreover, the middle band corresponding to *drpr-RA/RC* showed increased expression. These findings indicate that *drpr^CR1^* flies lack full-length *drpr* (encoding Drpr-I) and exhibit altered expression of Drpr-II and/or Drpr-III.

qRT-PCR of 7 dpe fly heads showed that *drpr^CR1^* did not reduce *drpr* mRNA; instead, transcript levels were significantly higher than in *ctrl1* (Fig. 7e). Since the primers were targeted to common areas shared by all isoforms, the RT-qPCR results likely reflect a compensatory upregulation of shorter isoforms in the absence of Drpr-I. To visualize how *drpr^CR1^* affected Drpr protein, we conducted Western blot analysis on head lysates from 7 dpe flies. We detected Drpr-I, Drpr-II, and Drpr-III in controls, along with lower molecular weight forms that may reflect proteolysis of Drpr (Fig. 7f). Consistent with our RT-PCR results, we failed to detect Drpr-I in *drpr^CR1^* flies, but saw increased levels of Drpr-III. Surprisingly, Drpr-II and Drpr-III were also detectable in *drpr^Δ5^* flies (Fig. 7f), suggesting this allele may not be a null, although nonspecific antibody recognition cannot be excluded. Notably, the putative proteolytic fragments were not detected in *drpr^CR1^* or *drpr^Δ5^* flies, suggesting that these fragments are derived from Drpr-I. As the antibody recognizing Drpr detects the C-terminus, we could not determine whether the N-terminal portion of Drpr (first 130 amino acids) was retained in *drpr^CR1^* flies (Fig. 7g). AlphaFold modeling predicted that wild-type Drpr has a large extracellular domain, a transmembrane region, and a cytoplasmic tail, whereas the *drpr^CR1^* protein is truncated shortly after the frameshift within the extracellular domain, eliminating the transmembrane domain and cytoplasmic tail (Fig. 7h). Collectively, these results demonstrate that *drpr^CR1^* is a splice-form-specific allele of *drpr*, lacking the full-length isoform(s) encoding Drpr-I.

### The *drpr^CR1^* allele uncouples ovarian defects from brain degeneration

**Fig. 8.**
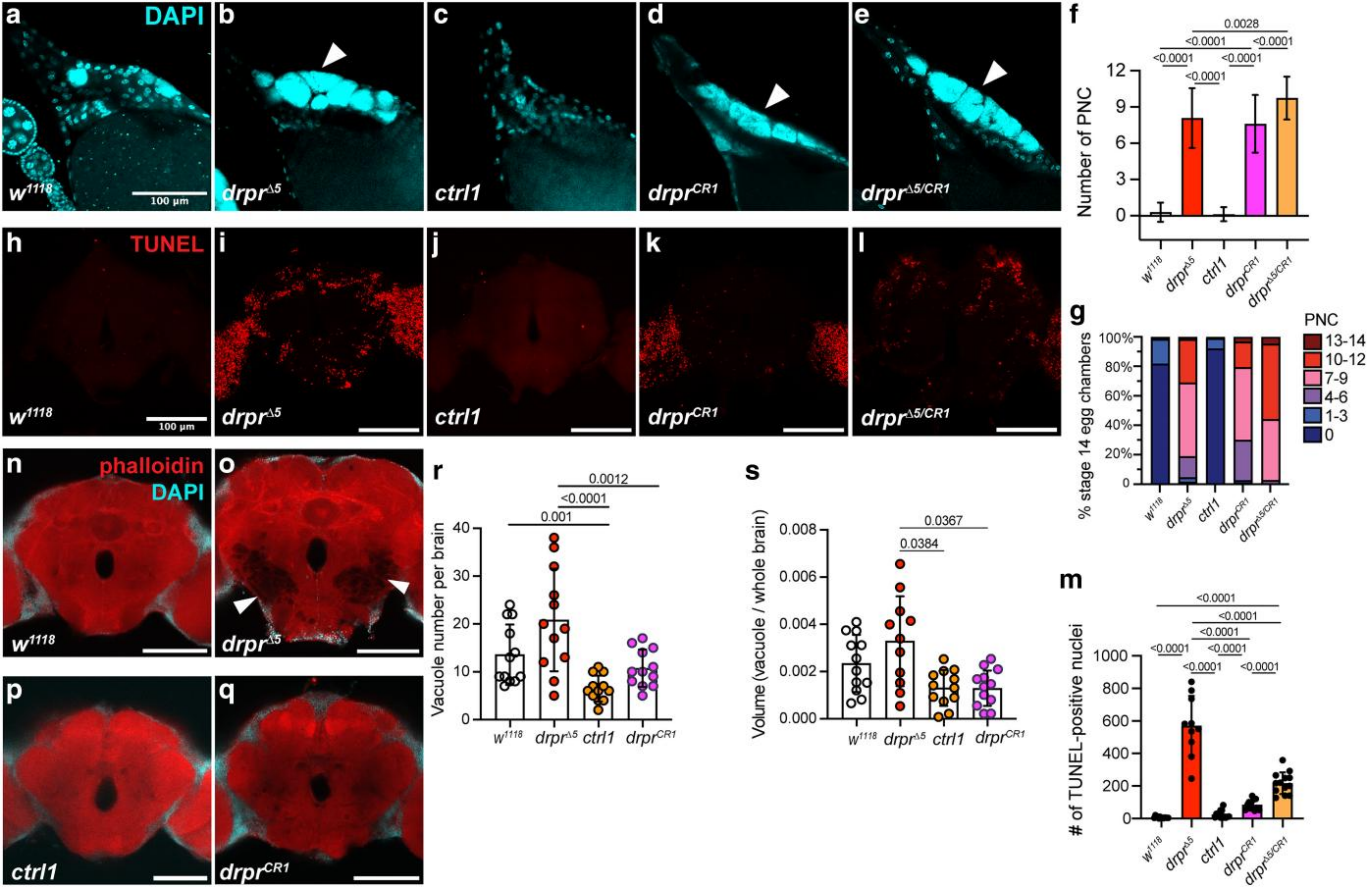
Ovarian and brain phenotypic analysis of the new *drpr* allele. a to e) Representative images of mature S14 egg chambers stained with DAPI showing PNC nuclei (white arrowheads) in the indicated genotypes (*w^1118^*, *drpr^Δ5^*, *ctrl1*, *drpr^CR1^*, and *drpr^Δ5/CR1^*). Scale bar, 100 µm. f) Quantification of PNC nuclei per egg chamber. *w^1118^*, *n* = 297; *drpr^Δ5^*, *n* = 132; *ctrl1*, *n* = 406; *drpr^CR1^*, *n* = 272; *drpr^Δ5/CR1^*, *n* = 146. Data are mean ± SD. Statistical significance was assessed using zero-inflated negative binomial model (R software). *P* < 0.05 was considered significant. g) Alternative quantification of data presented in Fig. 8f. h to l) Representative projections (30 µm thick) of anterior brain regions from 7-d-old adult, showing TUNEL-positive dead cells (red). Scale bar, 100 µm. m) Quantification of TUNEL-positive cells. Each point represents 1 brain; *w^1118^*, *n* = 13; *drpr^Δ5^*, *n* = 10; *ctrl1, n* = 14; *drpr^CR1^*, *n* = 12; *drpr^Δ5/CR1^*, *n* = 13. Data are mean ± SD. Zero-inflated negative binomial model was performed. n to q) Representative images of 40-d-old brains showing periesophageal neuropils stained with phalloidin (red) and DAPI (cyan). Vacuoles are indicated by white arrowheads. Scale bar, 100 µm. Brain region nomenclature follows (Ito et al. 2014). r to s) Quantification of vacuole number (r) and volume (s) in 40-d-old brains of flies with indicated genotypes. Each data point represents 1 brain, *n* = 12 per genotype. Negative binomial model was performed for vacuole number. One-way ANOVA with Dunnett's posttest was performed for vacuole volume (R software). *P* < 0.05 was considered significant.

We next examined the phenotypes of the *drpr^CR1^* allele in the ovary and brain. *drpr^CR1^* flies were homozygous viable and fertile. In the ovary, both *drpr^Δ5^* and *drpr^CR1^* lacked detectable Drpr antibody staining (Supplementary Fig. 3a to d’). Similar to *drpr^Δ5^*, *drpr^CR1^* exhibited a significant increase in PNC nuclei per S14 egg chamber compared with their corresponding controls, with no significant difference between the 2 alleles (Fig. 8a to g). Interestingly, trans-heterozygous flies (*drpr^Δ5/CR1^*) showed a stronger phenotype (Fig. 8e to g).

In the brain, both *drpr^Δ5^* and *drpr^CR1^* also lacked detectable Drpr antibody staining (Supplementary Fig. 3e to h’), but their phenotypes diverged. Analysis of persisting dead cells labeled by TUNEL in central brains (excluding optic lobes) revealed that both *drpr^Δ5^* and *drpr^CR1^* had significantly more dead cells than their respective controls, but the number was significantly lower in *drpr^CR1^* than in *drpr^Δ5^* (Fig. 8h to m). *drpr^Δ5/CR1^* flies exhibited significantly more TUNEL-positive signals than *drpr^CR1^* mutants but fewer than *drpr^Δ5^* mutants, indicating that the CRISPR allele partially complements the *drpr^Δ5^* mutation in the brain (Fig. 8l and m). Likewise, both the number and volume of neurodegenerative vacuoles associated with neuronal death were comparable between *drpr^CR1^* and *ctrl1*, but significantly reduced relative to *drpr^Δ5^* (Fig. 8n and s).

Together, these findings suggest that Drpr-I is indispensable for ovarian function but less critical for brain integrity, thereby indicating that Drpr functions differently in these 2 tissues. Alternatively, the overexpression of Drpr-III may lead to differential effects in these tissues. Future studies exploring the contributions of Drpr-I and Drpr-III in different tissues may resolve this discrepancy.

## Discussion

This study provides a systematic comparison of *drpr^RNAi^* constructs and mutant alleles in both the ovary and brain, revealing key differences in knockdown efficiency and phenotypic outcomes. All tested RNAi lines effectively eliminated Drpr protein by immunostaining, yet qRT-PCR showed variable transcript reductions, consistent with reports that RNAi efficiency depends on hairpin design, driver strength, and tissue context (Mohr et al. 2014). The SH RNAi construct in the VALIUM20 vector proved more effective in glial cells, correlating with stronger neurodegenerative phenotypes, while the LH construct abolished Drpr protein without significantly reducing mRNA, suggesting translational inhibition. These findings underscore the importance of validating RNAi knockdown at both protein and transcript levels.

Phenotypically, both ovarian and brain assays confirmed Drpr's essential role in cell clearance. In the ovary, both RNAi lines and mutant alleles produced PNC nuclei, establishing this as a robust readout of Drpr loss of function. In the brain, glial-specific RNAi produced persisting TUNEL-positive cells and neurodegenerative vacuoles, with severity depending on construct type and driver system. Interestingly, the spatial distribution of vacuoles differed between UAS/GAL4 and QUAS/QF2 systems, suggesting that not only RNAi design but also driver properties influence neurodegenerative outcomes.

The newly generated *drpr^CR1^* allele offers insights into isoform-specific functions. Unlike the widely used *drpr^Δ5^* allele, which disrupts the promoter and adjacent gene, *drpr^CR1^* specifically deletes exons 5 to 6, abolishing Drpr-I while preserving shorter isoforms. Strikingly, *drpr^CR1^* phenocopied *drpr^Δ5^* in the ovary but displayed milder neurodegeneration in the brain, demonstrating that Drpr-I is indispensable for follicle cell-mediated clearance but less critical in glia. RT-PCR and Western blot analyses revealed upregulation of Drpr-III and retention of Drpr-II in *drpr^CR1^* flies, consistent with isoform compensation. These shorter isoforms may retain partial phagocytic or signaling activity in neural tissue despite lacking the canonical full-length extracellular domain. Indeed, glial knockdown of Drpr-I alone results in persisting cell corpses, but at lower levels than the pan-isoform knockdown (Etchegaray et al. 2016). The ovarian requirement for Drpr-I may reflect unique structural demands of large-scale cell clearance during oogenesis or a lack of redundancy in this tissue. Another possibility is a differential requirement for the bridging molecule Orion, which binds the extracellular domain of Drpr to facilitate apoptotic cell recognition in the larval brain (Ji et al. 2023). Although the precise Orion binding site on Drpr remains unknown, its presence in the brain but potentially limited function in the ovary could explain the tissue-specific requirement for Drpr-I.

Together, these findings resolve ambiguities surrounding *drpr* alleles and RNAi tools. They highlight that while RNAi-mediated knockdown reliably recapitulates *drpr* loss of function, construct- and tissue-specific variability must be carefully considered in experimental design. Moreover, the *drpr^CR1^* allele uncouples ovarian and brain phenotypes, establishing a powerful tool to dissect isoform- and domain-specific roles of Drpr. Future studies should explore whether Drpr-II and Drpr-III engage distinct signaling pathways to support glial function and how tissue-specific demands shape Drpr isoform utilization.

## Supplementary Material

jkag040_Supplementary_Data

## Data Availability

Strains are available upon request. The authors affirm that all data necessary for confirming the conclusions of the article are present within the article, figures, and tables. Supplemental material available at *G3* online.

## References

[jkag040-B1] Awasaki T et al 2006. Essential role of the apoptotic cell engulfment genes *draper* and *ced-6* in programmed axon pruning during *Drosophila* metamorphosis. Neuron. 50:855–867. 10.1016/j.neuron.2006.04.027. 16772168

[jkag040-B2] Buchanan RL, Benzer S. 1993. Defective glia in the *Drosophila* brain degeneration mutant *drop-dead*. Neuron. 10:839–850. 10.1016/0896-6273(93)90200-B. 8494644

[jkag040-B3] Coombe PE, Heisenberg M. 1986. The structural brain mutant vacuolar medulla of *Drosophila melanogaster* with specific behavioral defects and cell degeneration in the adult. J Neurogenet. 3:135–158. 10.3109/01677068609106845. 3090215

[jkag040-B4] Coutinho-Budd JC, Sheehan AE, Freeman MR. 2017. The secreted neurotrophin spätzle 3 promotes glial morphogenesis and supports neuronal survival and function. Genes Dev. 31:2023–2038. 10.1101/gad.305888.117. 29138279 PMC5733495

[jkag040-B5] Draper I et al 2014. Silencing of DRPR leads to muscle and brain degeneration in adult *Drosophila*. Am J Pathol. 184:2653–2661. 10.1016/j.ajpath.2014.06.018. 25111228 PMC4188861

[jkag040-B6] Elguero JE et al 2023. Defective phagocytosis leads to neurodegeneration through systemic increased innate immune signaling. IScience. 26:108052. 10.1016/j.isci.2023.108052. 37854687 PMC10579427

[jkag040-B7] Etchegaray JI et al 2012. Draper acts through the JNK pathway to control synchronous engulfment of dying germline cells by follicular epithelial cells. Development. 139:4029–4039. 10.1242/dev.082776. 22992958 PMC3472587

[jkag040-B8] Etchegaray JI et al 2016. Defective phagocytic corpse processing results in neurodegeneration and can be rescued by TORC1 activation. Journal of Neuroscience. 36:3170–3183. 10.1523/JNEUROSCI.1912-15.2016. 26985028 PMC4792933

[jkag040-B9] Freeman MR, Delrow J, Kim J, Johnson E, Doe CQ. 2003. Unwrapping glial biology: Gcm target genes regulating glial development, diversification, and function. Neuron. 38:567–580. 10.1016/S0896-6273(03)00289-7. 12765609

[jkag040-B10] Goentoro LA, Yakoby N, Goodhouse J, Schüpbach T, Shvartsman SY. 2006. Quantitative analysis of the GAL4/UAS system in *Drosophila* oogenesis. Genesis (United States). 44:66–74. 10.1002/gene.20184. 16425298

[jkag040-B11] Han C et al 2014. Epidermal cells are the primary phagocytes in the fragmentation and clearance of degenerating dendrites in *Drosophila*. Neuron. 81:544–560. 10.1016/j.neuron.2013.11.021. 24412417 PMC3995171

[jkag040-B12] Hu Y et al 2013. FlyPrimerBank: an online database for *Drosophila melanogaster* gene expression analysis and knockdown evaluation of RNAi reagents. G3 (Bethesda). 3:1607–1616. 10.1534/g3.113.007021. 23893746 PMC3755921

[jkag040-B13] Ito K et al 2014. A systematic nomenclature for the insect brain. Neuron. 81:755–765. 10.1016/j.neuron.2013.12.017. 24559671

[jkag040-B14] Jenkins VK, Larkin A, Thurmond J. 2022. Using FlyBase: a database of *Drosophila* genes and genetics. In Methods in Molecular Biology. 2540:1–34. 10.1007/978-1-0716-2541-5_1. 35980571

[jkag040-B15] Ji H et al 2023. The *Drosophila* chemokine-like Orion bridges phosphatidylserine and draper in phagocytosis of neurons. Proc Natl Acad Sci U S A. 120:e2303392120. 10.1073/pnas.2303392120. 37276397 PMC10268242

[jkag040-B16] Kretzschmar D, Hasan G, Sharma S, Heisenberg M, Benzer S. 1997. The Swiss cheese mutant causes glial hyperwrapping and brain degeneration in *Drosophila*. Journal of Neuroscience. 17:7425–7432. 10.1523/jneurosci.17-19-07425.1997. 9295388 PMC6573436

[jkag040-B17] Lai SL, Lee T. 2006. Genetic mosaic with dual binary transcriptional systems in *Drosophila*. Nat Neurosci. 9:703–709. 10.1038/nn1681. 16582903

[jkag040-B18] Li H et al 2022. Fly cell atlas: a single-nucleus transcriptomic atlas of the adult fruit fly. Science. 375:eabk2432. 10.1126/science.abk2432. 35239393 PMC8944923

[jkag040-B19] Liu G, Bandyadka S, McCall K. 2024. Protocol to analyze 3D neurodegenerative vacuoles in *Drosophila melanogaster*. STAR Protoc. 5:103017. 10.1016/j.xpro.2024.103017. 38635393 PMC11043950

[jkag040-B20] Logan MA et al 2012. Negative regulation of glial engulfment activity by draper terminates glial responses to axon injury. Nat Neurosci. 15:722–730. 10.1038/nn.3066. 22426252 PMC3337949

[jkag040-B21] MacDonald JM et al 2006. The *Drosophila* cell corpse engulfment receptor draper mediates glial clearance of severed axons. Neuron. 50:869–881. 10.1016/j.neuron.2006.04.028. 16772169

[jkag040-B22] Manaka J et al 2004. Draper-mediated and phosphatidylserine-independent phagocytosis of apoptotic cells by *Drosophila* hemocytes/macrophages. J Biol Chem. 279:48466–48476. 10.1074/jbc.M408597200. 15342648

[jkag040-B23] Mohr SE, Smith JA, Shamu CE, Neumüller RA, Perrimon N. 2014. RNAi screening comes of age: improved techniques and complementary approaches. Nat Rev Mol Cell Biol. 15:591–600. 10.1038/nrm3860. 25145850 PMC4204798

[jkag040-B24] Perkins LA et al 2015. The transgenic RNAi project at Harvard medical school: resources and validation. Genetics. 201:843–852. 10.1534/genetics.115.180208. 26320097 PMC4649654

[jkag040-B25] Port F, Chen HM, Lee T, Bullock SL. 2014. Optimized CRISPR/Cas tools for efficient germline and somatic genome engineering in *Drosophila*. Proc Natl Acad Sci U S A. 111:E2967–E2976. 10.1073/pnas.1405500111. 25002478 PMC4115528

[jkag040-B26] Rivera AJ et al 2025. Traffic Jam activates the Flamenco piRNA cluster locus and the Piwi pathway to ensure transposon silencing and *Drosophila* fertility. Cell Rep. 44:115354. 10.1016/j.celrep.2025.115354. 40209716 PMC12094058

[jkag040-B27] Ryder E et al 2004. The DrosDel collection: a set of P-element insertions for generating custom chromosomal aberrations in *Drosophila melanogaster*. Genetics. 167:797–813. 10.1534/genetics.104.026658. 15238529 PMC1470913

[jkag040-B28] Serizier SB, Peterson JS, McCall K. 2022. Non-autonomous cell death induced by the Draper phagocytosis receptor requires signaling through the JNK and SRC pathways. J Cell Sci. 135:jcs250134. 10.1242/jcs.250134. 36177600 PMC10658789

[jkag040-B29] Timmons AK et al 2016. Phagocytosis genes nonautonomously promote developmental cell death in the *Drosophila* ovary. Proc Natl Acad Sci U S A. 113:E1246–E1255. 10.1073/pnas.1522830113. 26884181 PMC4780630

[jkag040-B30] Wittmann CW, et al 2001. Tauopathy in *Drosophila*: neurodegeneration without neurofibrillary tangles. Science. 293:711–714. 10.1126/science.1062382. 11408621

[jkag040-B31] Ziegenfuss JS et al 2008. Draper-dependent glial phagocytic activity is mediated by Src and Syk family kinase signalling. Nature. 453:935–939. 10.1038/nature06901. 18432193 PMC2493287

[jkag040-B32] Zohar-Fux M et al 2022. The phagocytic cyst cells in *Drosophila* testis eliminate germ cell progenitors via phagoptosis. Sci Adv. 8:eabm4937. 10.1126/sciadv.abm4937. 35714186 PMC9205596

