# Supplementary material for: Comparative analysis of draper mutant alleles and RNAi expression systems in the ovary and brain of Drosophila melanogaster
Source: G3 (Bethesda). 2026 Feb 16;16(5):jkag040. doi: 10.1093/g3journal/jkag040 (PMC13148388; doi:10.1093/g3journal/jkag040)
Supplement: jkag040_Supplementary_Data [file jkag040_supplementary_data.zip › Supplemental_Figure_1_G3-2025-406280.pdf]

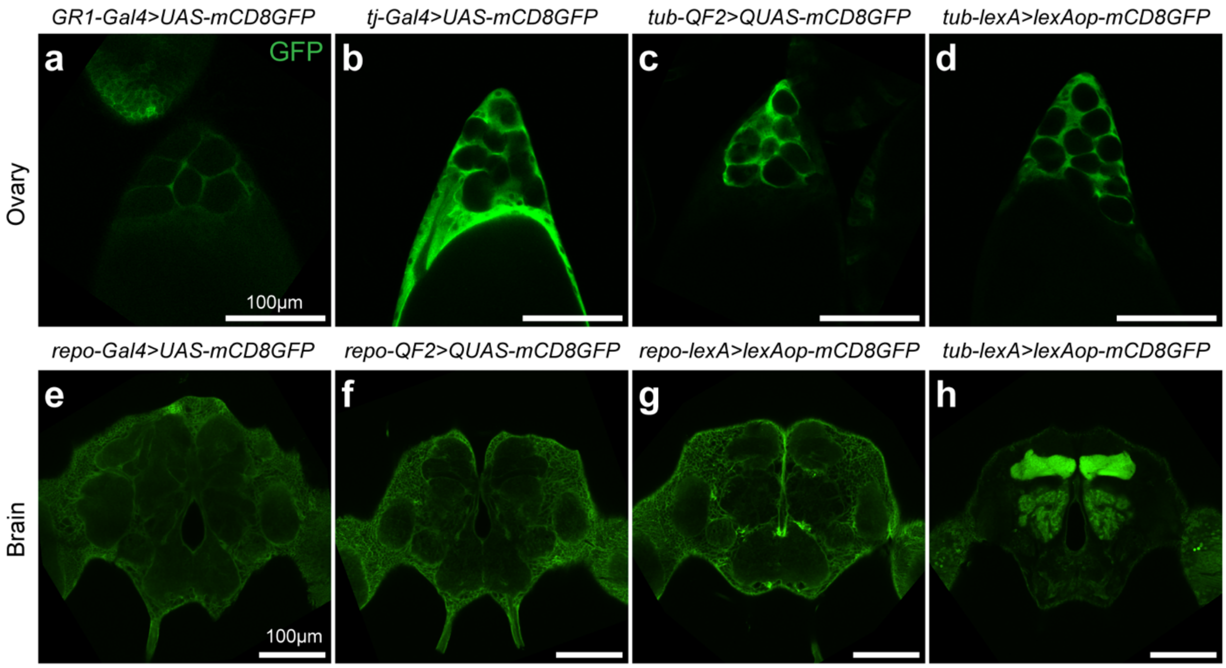

Figure S1. Visualization of driver expression pattern using mCD8::GFP.

(a-d) Expression pattern of *GR1-Gal4* (a), *tj-Gal4* (b), *tub-QF2* (c), and *tub-lexA* (d) in egg chambers. The anterior end of stage 12-13 egg chambers are shown to indicate expression in stretch follicle cells. (b-d) taken at same confocal settings but gain was increased in (a) because of lower expression in GR1.

(e-h) Expression pattern of *repoGal4* (e), *repo-QF2* (f), *repo-lexA* (g), and *tub-lexA* (h) in the brain.
