# Supplementary material for: Comparative analysis of draper mutant alleles and RNAi expression systems in the ovary and brain of Drosophila melanogaster
Source: G3 (Bethesda). 2026 Feb 16;16(5):jkag040. doi: 10.1093/g3journal/jkag040 (PMC13148388; doi:10.1093/g3journal/jkag040)
Supplement: jkag040_Supplementary_Data [file jkag040_supplementary_data.zip › Supplemental_Figure_2_G3-2025-406280.pdf]

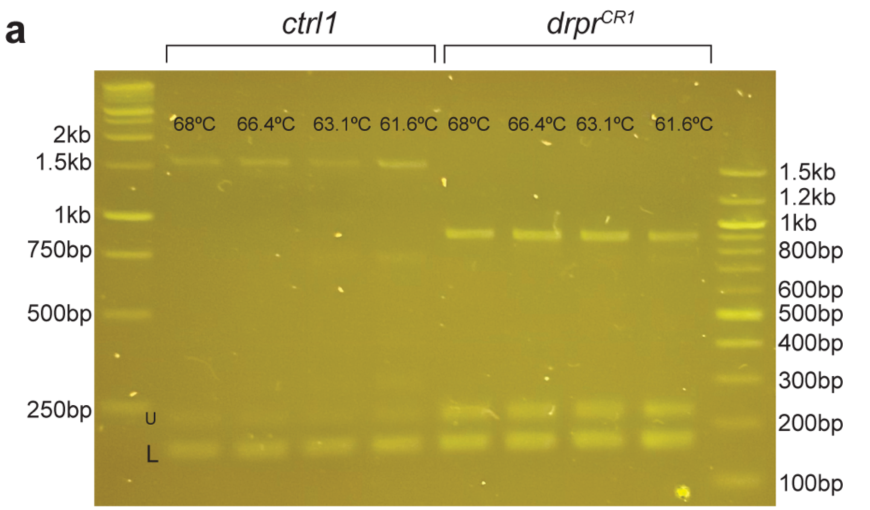

Expected transcript sizes:
wt transcript size of spliceforms *drpr-B, E, F* = 1.5kb
wt transcript size of spliceforms *drpr-A, C* = 168bp
wt transcript size of spliceforms *drpr-D* = 238bp
mutant transcript size if remainder of exon 6 is excluded = 224 bp

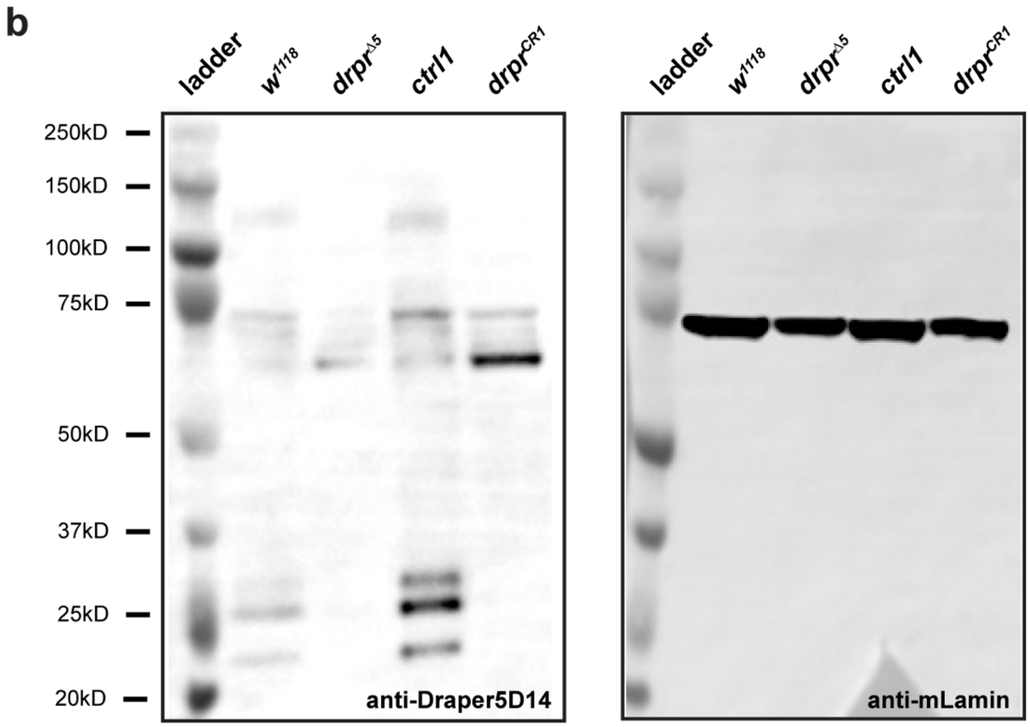

Figure S2. Full gel of RT-PCR products and western blot

(a) Full RT-PCR analysis of *ctrl1* and $drpr^{CR1}$ flies. Agarose gel showing PCR products spanning the mutation region.

(b) Full western blot of Drpr in head lysates from w^{1118}, $drpr^{\Delta 5}$, *ctrl1* and $drpr^{CR1}$ with Lamin as a loading control.
