# Supplementary material for: Comparative analysis of draper mutant alleles and RNAi expression systems in the ovary and brain of Drosophila melanogaster
Source: G3 (Bethesda). 2026 Feb 16;16(5):jkag040. doi: 10.1093/g3journal/jkag040 (PMC13148388; doi:10.1093/g3journal/jkag040)
Supplement: jkag040_Supplementary_Data [file jkag040_supplementary_data.zip › Supplemental_Figure_3_G3-2025-406280.pdf]

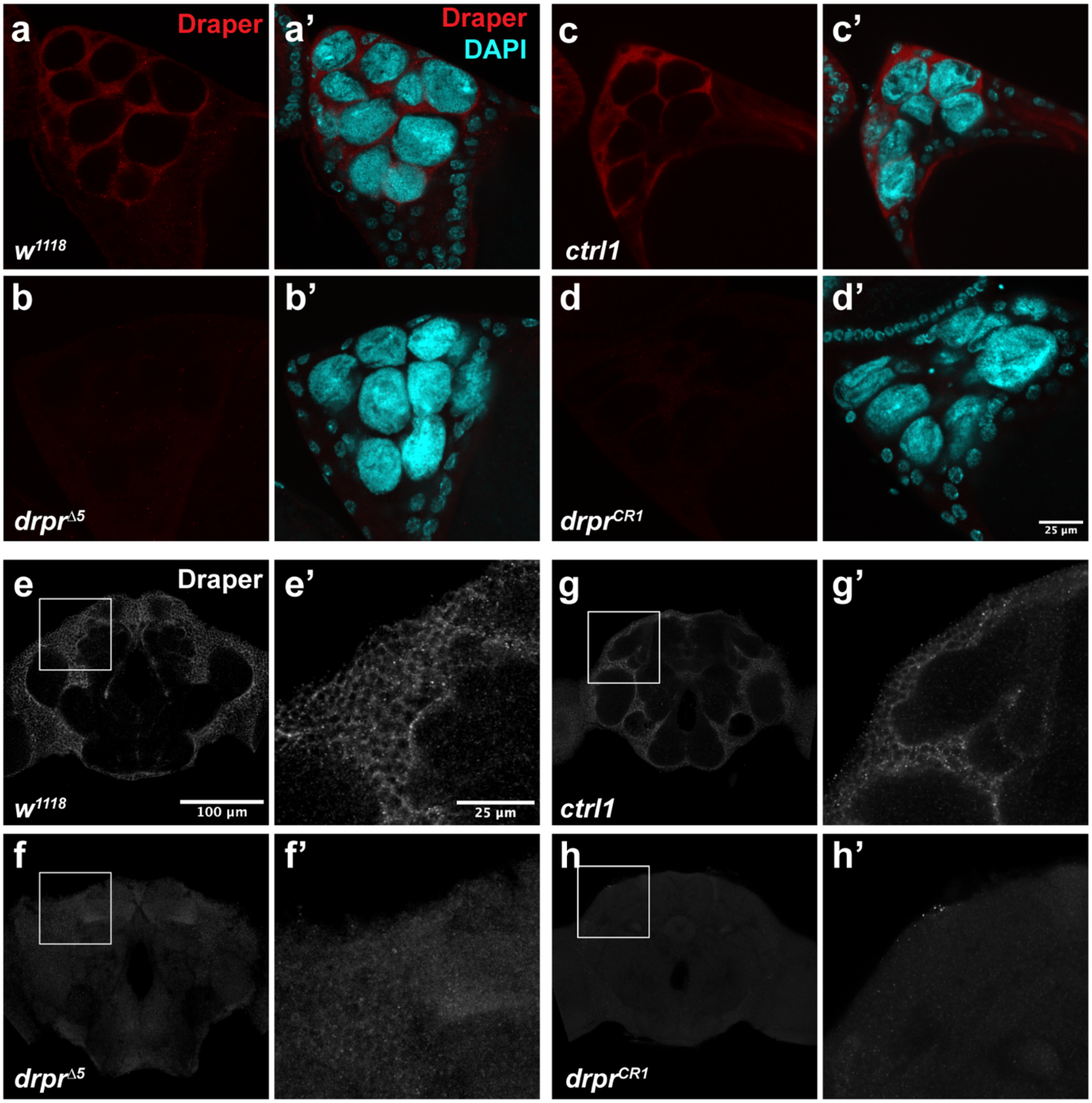

Figure S3. Drpr expression in the ovary and brain of *w*^{1118}, *drpr*$^{\Delta5}$, *ctrl1* and *drpr*^{CR1}

(a-d') Stage 14 egg chambers stained with Draper (Drpr, red) and DAPI (cyan) in the indicated genotypes (*w*^{1118}, *drpr*$^{\Delta5}$, *ctrl1* and *drpr*^{CR1}). Scale bar, 25 μm.

(e-h') Brains stained with Drpr (grey) in the indicated genotypes (*w*^{1118}, *drpr*$^{\Delta5}$, *ctrl1* and *drpr*^{CR1}). Main panel scale bar, 100 μm; zoom-in panels, 25 μm.
